# Two Novel, Distantly Related Papillomaviruses Isolated from Healthy Skin of the Timor Deer (Rusa timorensis)

**DOI:** 10.1128/genomeA.00505-18

**Published:** 2018-06-14

**Authors:** Beatriz Mengual-Chuliá, Gudrun Wibbelt, Marc Gottschling, Ignacio G. Bravo

**Affiliations:** a Infections and Cancer Laboratory, Catalan Institute of Oncology (ICO), Barcelona, Spain; b Department of Wildlife Diseases, Leibniz Institute for Zoo and Wildlife Research, Berlin, Germany; c Department Biologie I, Systematic Botany and Mycology, GeoBio-Center, Ludwig-Maximilians-Universität, Munich, Germany; d Centre National de la Recherche Scientifique (CNRS) Laboratory MIVEGEC (UMR CNRS IRD UM), Montpellier, France

## Abstract

We report the complete genome sequences of Rusa timorensis papillomavirus 1 (RtimPV1) and Rusa timorensis papillomavirus 2 (RtimPV2), isolated from hair follicles of asymptomatic skin from the same Timor deer specimen. RtimPV1 and RtimPV2 are evolutionarily only distantly related. RtimPV1 lacks a canonical E2-binding site, and RtimPV2 does not carry an *E6* gene.

## GENOME ANNOUNCEMENT

The genome of papillomaviruses (PVs) is a single double-stranded DNA (dsDNA) circular molecule of approximately 8 kbp, carrying genes involved in cell replication, viral replication, and viral encapsidation. Most PVs cause epithelial asymptomatic infections in amniotes and probably also in bony fishes, while some of them induce proliferative benign lesions and different cancers (1, 2).

The Timor deer Rusa timorensis (de Blainvillee, 1822) is native to the island of Java and is classified as “vulnerable” by the International Union for Conservation of Nature (IUCN) Red List of Threatened Species (http://www.iucnredlist.org) (3). A biopsy specimen from asymptomatic skin (isolate IZW 39/08) was collected from a female deer, about 19 years old, living in captivity in a zoo in Berlin, Germany. The animal did not present papilloma-like lesions and was euthanized because of a severe internal infection that was unrelated to any PV infection. Total DNA was extracted from hair bulbs and PCR tested for the presence of PV DNA using broad-spectrum primers targeting the *E1* and *L1* genes (4). Using this partial information, we designed tail-to-tail primers to amplify the full-length genomes using long-range PCR, which were then Sanger sequenced in both strands through primer walking and cloned. Phylogenetic relationships for the *E1E2*, *L2L1*, and *E1E2L2L1* gene concatenates were inferred under a maximum-likelihood framework (5).

We retrieved two complete PV genomes from the same skin sample of the *R. timorensis* individual, here named Rusa timorensis papillomavirus 1 (RtimPV1) (isolate IZW 39/08) and Rusa timorensis papillomavirus 2 (RtimPV2) (isolate IZW 39/08). Both viral genomes display the standard features of PVs, with a long control region; the early genes *E7*, *E1*, and *E2*; and the late genes *L2* and *L1*. Remarkably, RtimPV2 does not carry an *E6* gene. This gene may have been lost independently in several PV lineages through time (6, 7). The RtimPV1 *L1* gene displays maximum nucleotide similarity values around 65% with very divergent PVs, while RtimPV2 *L1* displays identity values around 65% with divergent PVs in the 5ʹ end, and only above 50% in the 3ʹ end. Therefore, this criterion alone does not suffice for taxonomic assignment following the current rules (8), which need to be revised (7, 9). Phylogenetic reconstruction confidently places RtimPV1 as a sister lineage to Capra hircus papillomavirus 1 (ChPV1), isolated from healthy skin of a female goat (10), a *Phipapillomavirus* in the Beta + Xi PV supergroup (11). Neither ChPV1 nor RtimPV1 displays a canonical E2-binding site in the long control region, thus suggesting alternative regulation of early gene expression. Phylogenetic inference unambiguously places RtimPV2 basal to *Epsilonpapillomavirus* and *Deltapapillomavirus*, within the Delta + Zeta PV supergroup, which includes PVs isolated from giraffes and camels, as well as from different bovid and deer species (11). The RaPV genome isolated from Rusa alfredi (Sclater, 1870), with a host closely related to R. timorensis, branches in a derived position within the Delta + Zeta PV supergroup (12) and is only distantly related to RtimPV2.

Our results exemplify the diversity of PVs asymptomatically infecting similar cells within a single host individual and highlight the genomic plasticity within Papillomaviridae.

### Accession number(s).

The complete genome sequences for RtimPV1 and for RtimPV2 are available in GenBank under the accession no. KP757765 and KT852571, respectively.
